# Iron uptake mediated by the plant-derived chelator nicotianamine in the small intestine

**DOI:** 10.1074/jbc.RA120.015861

**Published:** 2020-12-25

**Authors:** Yoshiko Murata, Masami Yoshida, Naho Sakamoto, Shiho Morimoto, Takehiro Watanabe, Kosuke Namba

**Affiliations:** 1 Bioorganic Research Institute, Suntory Foundation for Life Sciences, Soraku-gun, Kyoto, Japan; 2 Graduate School of Pharmaceutical Science, Tokushima University, Tokushima, Japan

**Keywords:** iron, intestine, transport metal, transporter, Xenopus, oocyte, nicotianamine, plant-derived chelator, Caco-2 cells, 5-HTP, 5-hydroxytryptophan, Dcytb, duodenal cytochrome b, DMT1, divalent metal transporter, FMOC, 9-fluorenylmethyloxycarbonyl, FPN1, ferroportin1, MAs, phytosiderophores, NA, nicotianamine, PAT1, proton-coupled amino acid transporter, PEPT1, oligopeptide transporter, TLC, thin layer chromatography, YS1/YSL, yellow stripe 1/yellow stripe like

## Abstract

Iron is an essential metal for all living organisms that is absorbed in the intestinal cells as a heme-chelated or free form. It is unclear how important plant-derived chelators, such as nicotianamine (NA), an organic small molecule that is ubiquitous in crops, vegetables, and various other foods, contribute to iron bioavailability in mammals. We performed electrophysiological assays with *Xenopus laevis* oocytes and radioactive tracer experiments with Caco-2 cells. The findings revealed that the proton-coupled amino acid transporter SLC36A1 (PAT1) transports iron in the form of NA-Fe (II) complex *in vitro*. Decreased expression of *hPAT1* by RNA interference in Caco-2 cells reduced the uptake of NA-^59^Fe (II) complex. The uptake of inorganic ^59^Fe (II) was relatively unaffected. These results imply that PAT1 transports iron as a NA-Fe (II) complex. The rate of ^59^Fe absorption in the spleen, liver, and kidney was higher when mice were orally administered NA-^59^Fe (II) compared with free ^59^Fe (II). The profile of site-specific *PAT1* expression in the mouse intestine coincided with those of NA and iron contents, which were the highest in the proximal jejunum. Orally administered NA-^59^Fe (II) complex in mice was detected in the proximal jejunum by thin layer chromatography. In contrast, much less ^59^Fe (or NA) was detected in the duodenum, where the divalent metal transporter SLC11A2 (DMT1) absorbs free Fe (II). The collective results revealed the role of PAT1 in NA-Fe (II) absorption in the intestine and potential implication of NA in iron uptake in mammals.

Iron is an essential element for almost all living organisms. This reflects its pivotal roles in diverse metabolic processes, including oxygen circulation, DNA synthesis, and the electron transport chain (1). Iron deficiency is the most prevalent global nutritional disorder. For example, anemia affects roughly a third of the world’s population, and half of the cases involve iron deficiency. Children, women of childbearing age, and pregnant women are particularly at risk. Thus, iron deficiency is a major global public health concern (2, 3).

Human dietary iron is typically classified as heme and nonheme iron (4). Heme iron is abundant in meat and is more efficiently absorbed in the small intestine than nonheme iron. Heme carrier protein 1 (HCP1) is a transporter protein. It was first described as a transporter of heme iron (5), but was subsequently shown to be a proton-coupled folate transporter (6). Thus, the processes and proteins functioning in heme iron absorption by mammals remain unclear.

In addition to inorganic iron, nonheme iron is present in diverse forms in both plant and animal foods. The low pH of the stomach can stabilize iron in the reduced form of Fe (II) or ferrous ion. Dietary iron is typically the oxidized Fe(III) or ferric ion, which has low water solubility and poor bioavailability (4). The absorption of inorganic iron mainly occurs across polarized intestinal epithelial cells in the duodenum and proximal jejunum (4). Dietary Fe(III) is reduced by duodenal cytochrome b (Dcytb) (7) and absorbed in the ferrous form, Fe(II), by the divalent metal ion transporter, solute carrier family 11 member 2 (SLC11A2; divalent cation transporter 1 [DCT1] or divalent metal transporter 1 [DMT1]), in the apical membrane of duodenal epithelial cells (8, 9). At that site, the absorbed iron is stored in the ferritin-bound form or is exported into the blood by the basolateral iron exporter SLC40A1 (ferroportin 1 [FPN1], iron-regulated transporter 1 [IREG1], or metal tolerance protein 1 [MTP1]) (10, 11, 12). Nonheme iron uptake in the duodenum is mediated exclusively by the Dcytb/DMT1 route. However, Dcytb knockout mice reportedly lack a particular phenotype, such as a decrease in hemoglobin levels, body weight, or liver and spleen iron contents (13). This observation indicates that there must be a mechanism independent of Dcytb/DMT1 activity for dietary Fe (II) absorption. Candidate iron chelators that facilitate Fe (II) absorption include citrate, ascorbic acid, and other dietary factors.

Humans ultimately depend on plants for their iron supply. Thus, the availability of plant iron is essential (14). Nicotianamine (NA) is a hexadentate metal chelator ubiquitously present in higher plants. NA is essential for iron translocation in plants (15, 16, 17) (Fig. 1*A*). NA is a precursor of the mugineic acid family phytosiderophores (MAs) (18, 19). It preferentially chelates Fe (II) but can also chelate Fe (III), in particular at higher pH levels. Therefore, NA acts as an intracellular iron scavenger to protect cells from iron-mediated oxidative damage (20). Membrane trafficking of NA-Fe(II) or MAs-Fe(III) complexes is mediated by yellow stripe 1/yellow stripe like (YS1/YSL) transporter proteins (21, 22, 23). These transporter proteins are members of the oligopeptide transporter family of proteins, some of which are involved in the transport of oligopeptide and amino acid derivatives (24, 25). NA-Fe (II) is essential in plant iron homeostasis. Edible plants including beans and vegetables and plant-based juices contain abundant NA (26). NA is also a potent enhancer of iron uptake by monolayer Caco-2 cells used as an intestinal model (27). However, the direct involvement of NA-Fe (II) complex in iron uptake in animals has yet to be experimentally proven. In addition, the mechanism of NA-Fe (II) complex uptake in the intestine is unknown.

**Figure 1:**
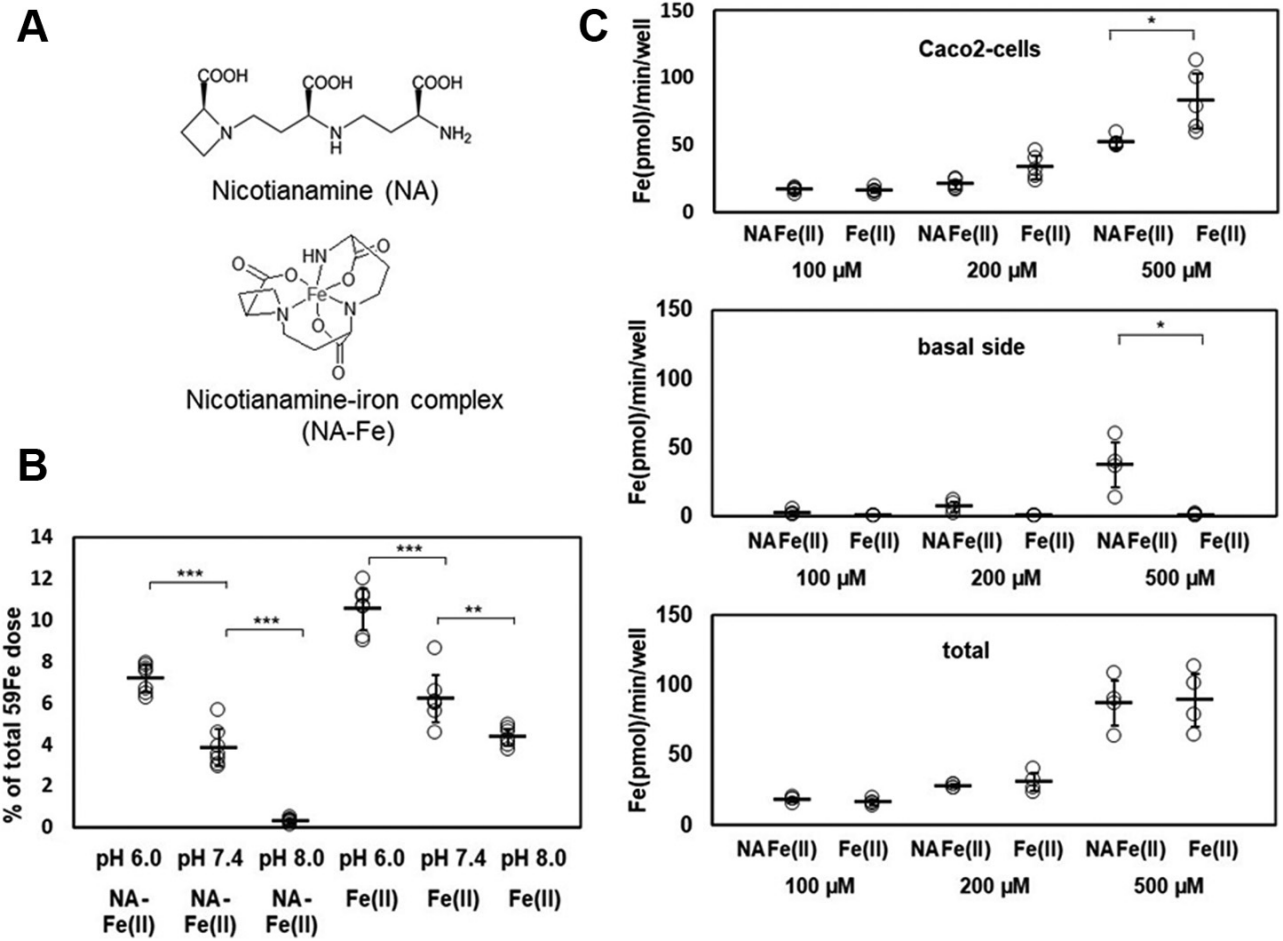
**NA-**^**59**^**Fe(II) transport in Caco-2 cell monolayers is pH-sensitive and concentration-dependent.** (*A*) structure of nicotianamine (NA) and the estimated coordination manner of NA–iron complex. (*B*) ^59^Fe radioactivity in the monolayer Caco-2 cells determined after the addition of 100 μM ^59^Fe(II) or NA-^59^Fe(II) containing 1 mM NA and 100 μM Fe(II) at pH 6.0, 7.4, or 8.0 (n = 7, ∗∗∗*p* < 0.001, ∗∗*p* < 0.01). (*C*) Caco-2 cells (n = 5) and the culture solution on the basal side (n = 4) were collected and counted 30 min after the addition of 100, 200, or 500 μM NA-^59^Fe(II) or ^59^Fe(II) (∗*p* < 0.05).

This study aimed to understand the mechanism of NA-mediated intestinal iron uptake, by identifying the NA-Fe (II) complex transporter in the intestine. The findings demonstrated that the SLC36A1 amino acid transporter (the human ortholog of proton-coupled amino acid transporter, hPAT1) (28), which belongs to the membrane-bound SLC proteins in human small intestine (29, 30), can facilitate the transport of NA and NA–iron complexes. These results implicate NA as having a pivotal role in iron trafficking in the mammalian intestine.

## Results

### Caco-2 monolayer cells display pH-sensitive and NA-Fe (II) concentration-dependent increases in iron uptake

We measured the uptake rates of 100 μM NA-Fe(II) and free Fe(II) containing 1 to 2% ^59^Fe(II) by Caco-2 monolayer cells to assess iron translocation efficiency at pH 6.0, 7.4, and 8.0 (Fig. 1*B*). To minimize the effect of DMT1, which transports Fe(II) (8) independent of NA, the iron uptake assays were performed in the presence of excess NA (NA/Fe(II) = 10:1). NA-^59^Fe (II) uptake was pH-dependent in the order pH 6.0 > pH 7.4 > pH 8.0 (Fig. 1*B*). The findings indicated that Caco-2 cells take up NA-Fe (II) using the proton gradient across the plasma membrane. This result is similar to that reported for the plant YS1/YSL transporters, which mediate the membrane trafficking of NA-Fe(II) complexes (23, 31). On the other hand, ^59^Fe efflux from the basal sides in NA-Fe (II) form was much higher than that in Fe (II) form, while the iron content within Caco-2 cells in NA-Fe (II) form was slightly lower than that in Fe (II) form, particularly at the substrate concentration of 500 μM (Fig. 1*C*). At each concentration, the total amounts of the intracellular and basal side ^59^Fe were comparable between NA-Fe (II) and Fe (II) (Fig. 1*C*). Previous studies revealed that DMT1 responsible for Fe(II) uptake by Caco2 cells negligibly transports iron in chelate forms (32). We obtained the same result for NA-Fe (II), as described below. Thus, the result in Figure 1*C* indicates that Fe (II) in the NA-Fe (II) form is taken up by an unknown transporter other than DMT1.

To confirm that NA and ^59^Fe were simultaneously taken up by Caco-2 cells, the cells were extracted 30 min after administration of NA-^59^Fe (II) and analyzed by cellulose thin layer chromatography (TLC) (Fig. S1). Both synthetic NA and NA-Fe (II) yielded single bands at an Rf value of approximately 0.7 following ninhydrin staining, which detects amino groups (Fig. S1*A*). ^59^Fe in the NA-^59^Fe (II) form and the free form were detected by autoradiography (Fig. S1*B*). The cell extract displayed a spot at the same Rf value (0.67) that agreed with that of authentic NA-^59^Fe(II) (Fig. S1, *C***–***D*). Since the free ^59^Fe remained at the origin on the TLC plate, the iron band visible at Rf 0.67 in the cell extract was thought to be the NA-^59^Fe(II) complex.

### NA and NA-Fe (II) are transported by hPAT1 expressed in *Xenopus laevis* oocytes

**Figure 2:**
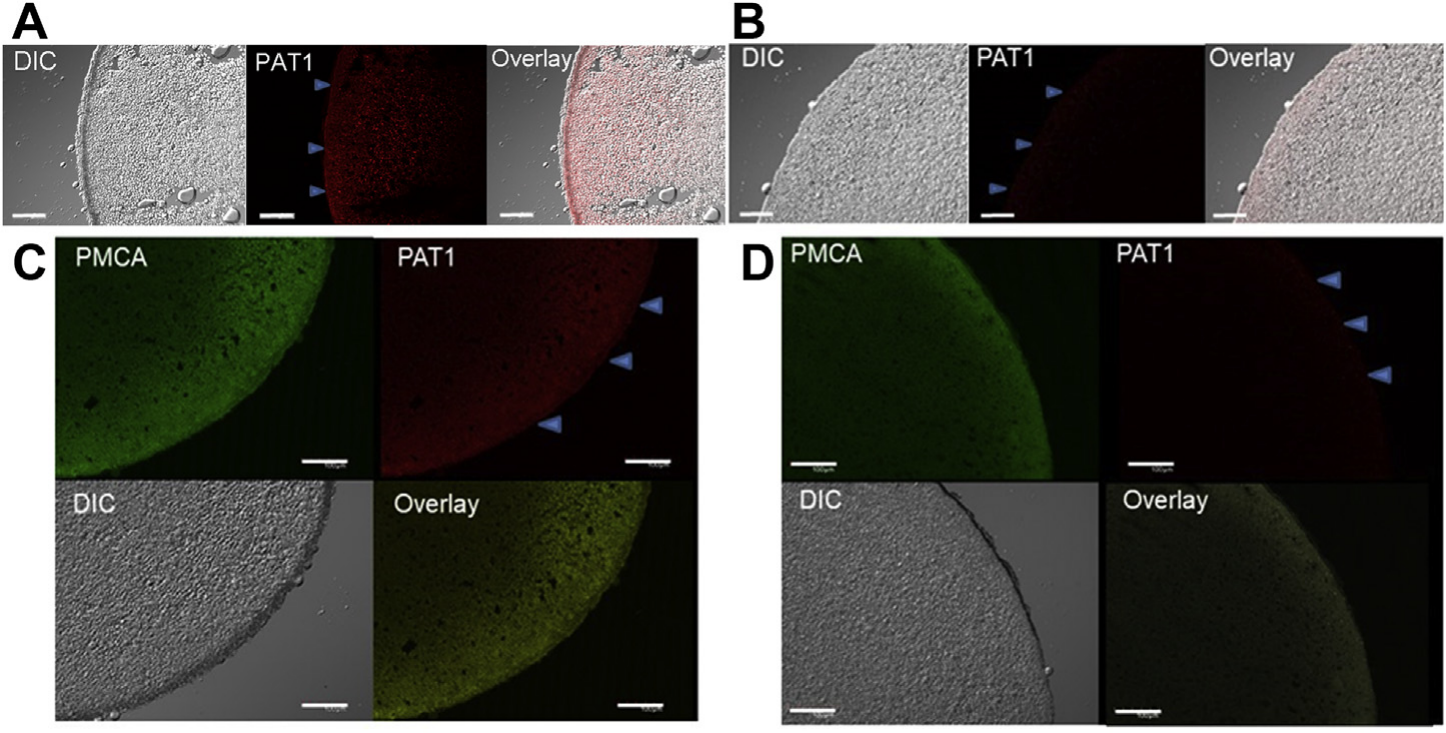
**hPAT1 expression in oocytes detected by immunohistochemistry using anti-hPAT1 polyclonal antibody staining.** (*A*) and (*C)*, hPAT1 expression (*red*) was detected using anti-hPAT1 antibody on the plasma membranes of oocytes (*C*) injected with *hPAT1* cRNA. (*B*) and (*D*), negative control (injected with water). (*C*-*D*) simultaneous immunostaining (*green*) with the plasma membrane marker Ca^2+^ ATPase (PMCA) was performed. The outer surfaces of the oocytes are indicated by *blue arrows*. DIC, differential interference contrast. Scale bar indicates 100 μm.

To identify the transporter responsible for the uptake of the NA-Fe (II) complex in the small intestine, we examined hPAT1, because NA could be regarded as an amino acid derivative (Fig. 1*A*). PAT comprises four subtypes, PAT1–PAT4 (30). RT-PCR analysis of human intestine and Caco-2 cells revealed that only the primer targeting *hPAT1* yielded a product (28) (Fig. S2, *A***–***B*). Therefore, we focused on hPAT1 as the candidate transporter for NA or NA-Fe(II) in the human intestine as well as Caco-2 cells (28). We injected *X. laevis* oocytes with *hPAT1* cRNA. Immunohistochemistry confirmed that hPAT1 was present on the plasma membranes of *hPAT1*-injected oocytes (Fig. 2, *A* and *C*) but not in control oocytes (Fig. 2, *B* and *D*). To show that hPAT1 was specifically localized in the plasma membrane, immunostaining was simultaneously performed using the plasma membrane marker Ca^2+^ ATPase (PMCA). Colocalization of hPAT1 and PMCA was observed (Fig. 2*C*). In control oocytes lacking hPAT1, only PMCA was detected, as expected (Fig. 2*D*).

**Figure 3:**
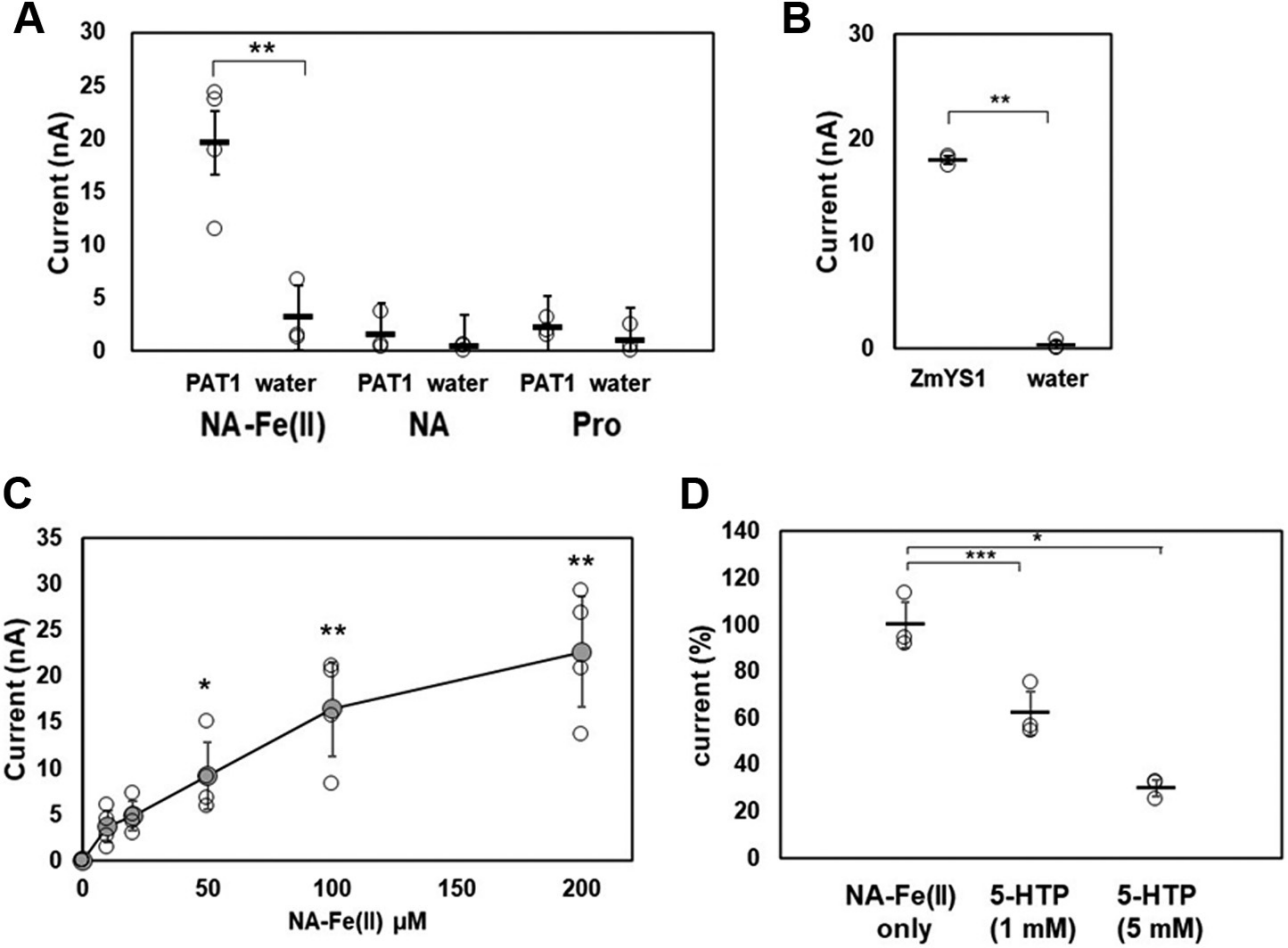
**Electrophysiological NA-**^**59**^**Fe(II) transport assays with *Xenopus* oocytes expressing hPAT1.** An oocyte was voltage-clamped at −60 mV during the current recording while the ND96 buffer (pH 6.0) was perfused. (*A*) iron transport activities of oocytes injected with *hPAT1* cRNA or water were measured in 100 μM NA-Fe(II) (1 mM NA:100 μM Fe), 100 μM NA or proline (Pro) (n = 6). (*B*) as a positive control of NA-Fe(II) transporter activity, *ZmYS1* cRNA or water was injected into oocytes, and the currents were measured in the presence of 100 μM NA-Fe(II). (*C*) currents induced by various concentrations of NA-Fe(II) (0, 10, 20, 50, 100, and 200 μM) (n = 3–6, ∗*p* < 0.05; ∗∗*p* < 0.01). (*D*) inhibitory effects of 1 or 5 mM 5-hydroxytryptophan (5-HTP) against NA-Fe(II)-elicited current were evaluated for 0.5 mM NA-Fe(II) (n = 3–5, ∗∗∗*p* < 0.001, ∗*p* < 0.05).

Electrophysiological assays next revealed that the hPAT1-expressing oocytes more effectively transported NA-Fe(II) (NA/Fe molar ratio =10:1) than NA or proline (33) (Fig. 3*A* and Fig. S3). The oocytes expressing ZmYS1, which was first identified as an NA-Fe(II) transporter in corn (21), showed almost the same activity as those expressing hPAT1 upon addition of NA-Fe(II) (Fig. 3*B*). A concentration-dependent current was generated upon the addition of 0 to 200 μM NA-Fe (II) (Fig. 3*C*). Based on these results, we estimated *K*_m_ of hPAT1 for the NA-Fe (II) complex to be 49.4 μM, which was significantly higher than that for proline. A *K*_m_ of hPAT1 for proline of 1.7 mM was reported in Caco-2 cells transfected with hPAT1 (28). In the presence of 1 mM and 5 mM of the PAT1-specific inhibitor 5-hydroxytryptophan (5-HTP) (34), NA-Fe(II)-induced current was reduced to 62.1% and 30.1% of the original levels, respectively (Fig. 3*D*).

NA and NA-Fe(II) transporter families in plants have been categorized as oligopeptide transporters, which are widely distributed in bacteria, archaea, and fungi (24, 25). Therefore, we analyzed human oligopeptide transporters using electrophysiological assays. Oocytes expressing the oligopeptide transporter hPEPT1 (SLC15A1) failed to transport either NA or NA-Fe(II), but were able to transport the positive control glycylsarcosine, which is a substrate for hPEPT1 (35) (Fig. S4*A*). As the next candidate, we examined the Fe (II) transporter hDMT1 (SLC11A2). It transported Fe (II) (100 μM) but not NA-Fe (II) (1 mM NA: 100 μM Fe), indicating that hDMT1 does not transport NA-Fe (II) as effectively as it transports Fe (II) (Fig. S4*B*).

### Knockdown of hPAT1 in Caco-2 cells decreases NA-Fe (II) uptake

**Figure 4:**
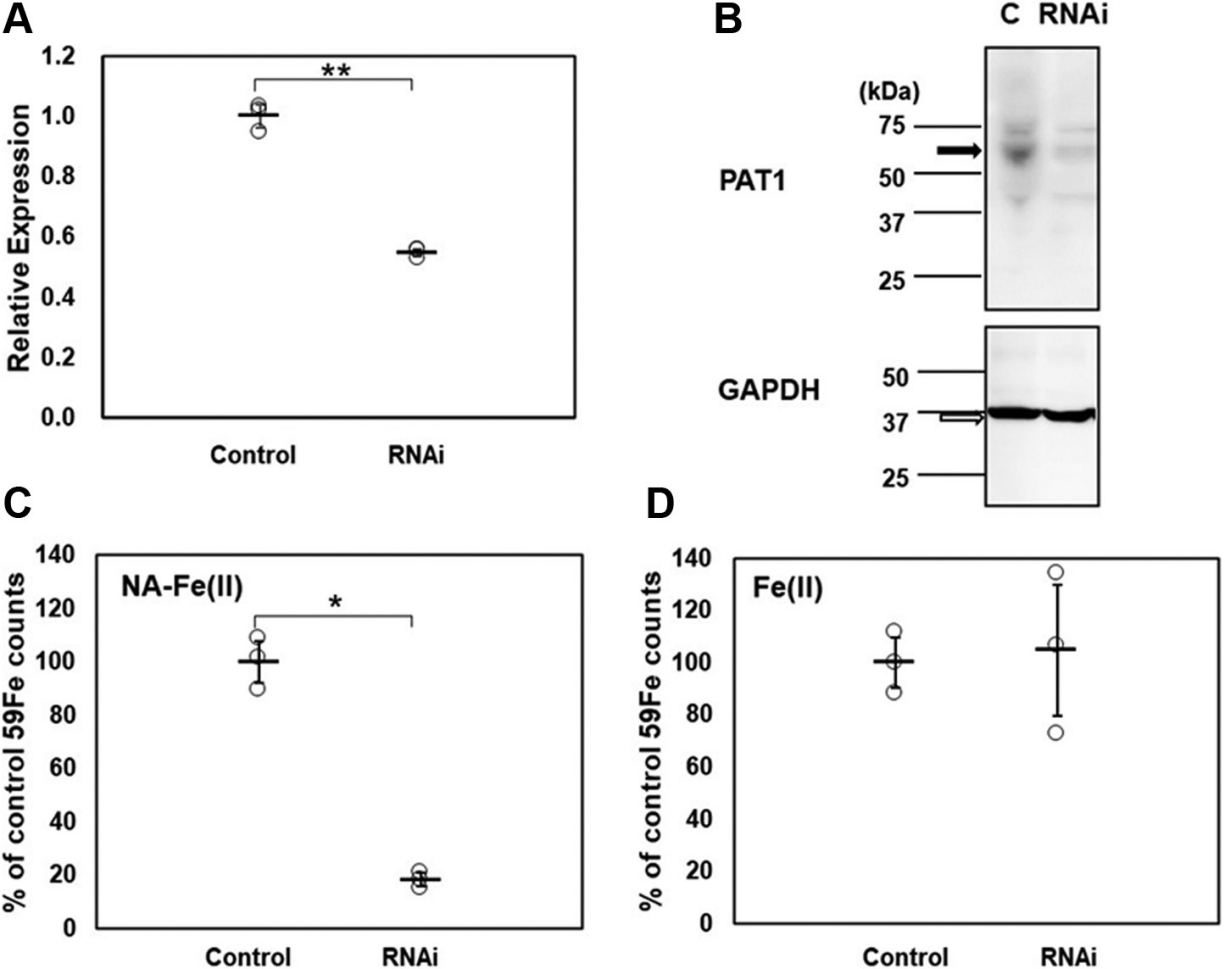
**Iron uptake in *hPAT1*-knockdown Caco-2 cells.** (*A*) *hPAT1* mRNA expression in RNAi-mediated *hPAT1*-knockdown Caco-2 cells (*dotted bars*) was measured by qRT-PCR. The control cells (*white bars*) were transformed with a Neg-miRNA plasmid (n = 3, ∗*p* < 0.05, ∗∗*p* < 0.01). (*B*) western blotting of Caco-2 cells transfected with plasmid only (column c) and *hPAT1* RNAi showing hPAT1 and GAPDH levels. The calculated molecular weights of PAT1 and GAPDH are about 53 kDa and 36 kDa respectively. The images were obtained using an Amersham Imager 600. (*C*) ^59^Fe isotope contents (as percentage of the control value) detected in *hPAT1*-knockdown Caco-2 cells, the radioactivity was measured after the addition of 100 μM NA-^59^Fe(II) (n = 3, ∗*p* < 0.05). (*D*) ^59^Fe isotope contents detected in *hPAT1*-knockdown Caco-2 cells; radioactivity was measured after the addition of free ^59^Fe(II) (100 μM) (n = 3).

To further confirm the NA-Fe (II) transport activity of hPAT1, we knocked down *hPAT1* in Caco-2 cells by RNA interference (RNAi). Caco-2 cells were transfected with three different RNAi sequences targeting nucleotides (463–483, 907–927, or 1608–1628) of the 5786-bp *hPAT1* mRNA (28). Among the three groups, only the cells transfected with the RNAi targeting nucleotides 463 to 483 of *hPAT1* grew to form monolayers. The expression of *hPAT1* was decreased to 55.0% of the level observed in control cells transfected with vector alone (Fig. 4*A*). Immunoblotting for hPAT1 demonstrated that *hPAT1* RNAi reduced hPAT1 protein levels by approximately 58% in Caco-2 cells relative to control cells (Fig. 4*B*). ^59^Fe(II)-NA uptake decreased to 18.3% in Caco-2 cells transfected with *hPAT1* RNAi (Fig. 4*C*), while free ^59^Fe(II) uptake was not significantly affected (Fig. 4*D*).

### Tissue specificity of iron distribution after oral administration of Fe (II) and NA-Fe (II)

**Figure 5:**
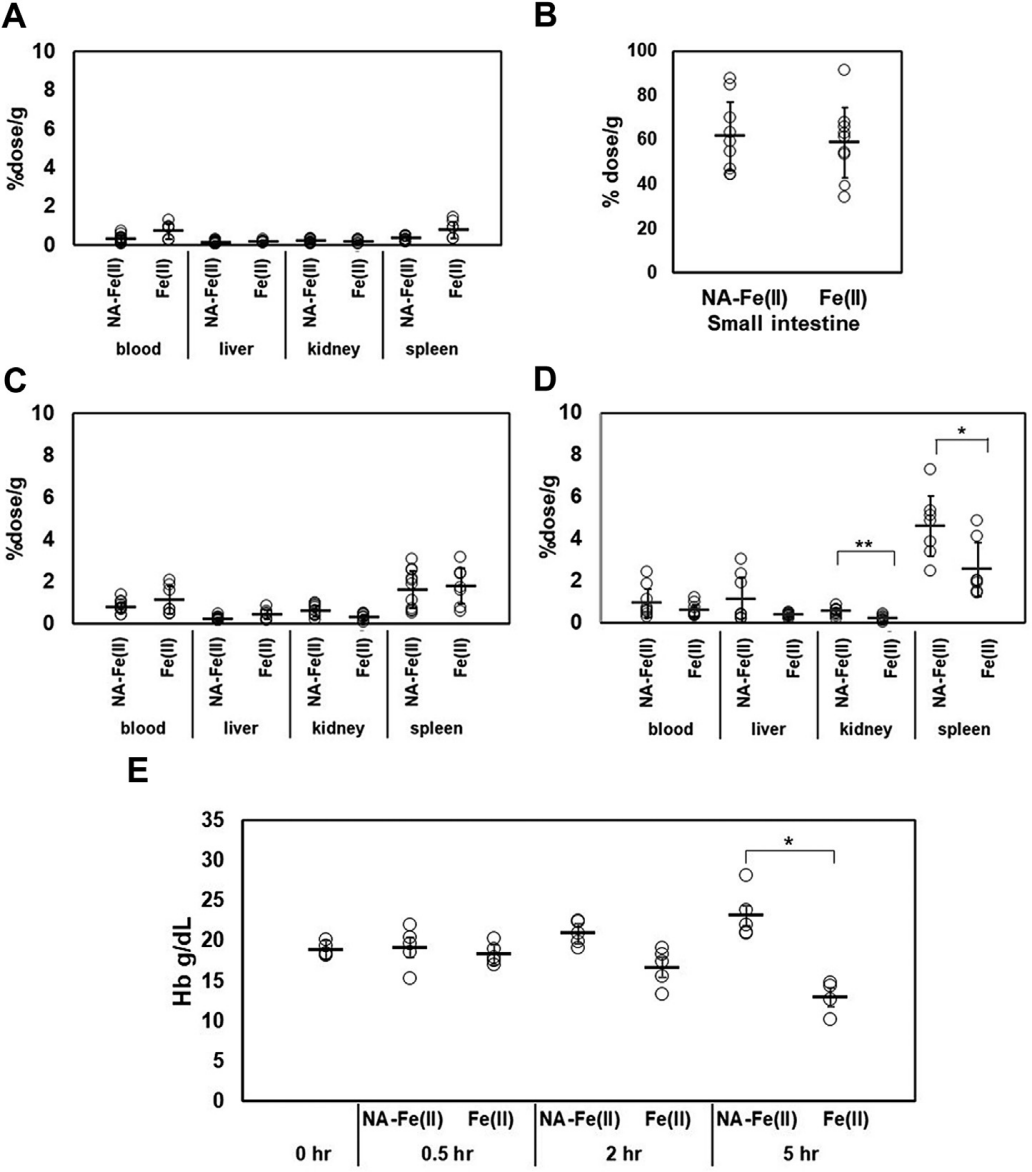
**Comparison of**^**59**^**Fe and Hb contents after oral administration of Fe and NA-Fe(II).**^59^Fe radioactivity was measured in the blood, liver, kidney, spleen (*A*), and small intestine at 0.5 h (*B*), 2 h (*C*), and 5 h (*D*) after oral administration of NA-^59^Fe(II) or ^59^Fe(II) (n = 7–9, ∗*p* < 0.05, ∗∗*p* < 0.01). The vertical axis is ^59^Fe counts for each organ per gram weight that was expressed as the percentage of total activity of ^59^Fe administered to mice. (*E*) hemoglobin (Hb) levels in whole blood of mouse were measured at 0 h, 0.5 h, 2 h, and 5 h after oral administration of NA-^59^Fe(II) or ^59^Fe(II) (n = 4, ∗*p* < 0.05).

We carried out *in vivo* experiments using mice to assess the assimilation of iron in the presence of NA. To eliminate the effects of diet-derived NA, 7-week-old male mice were bred for 1 week on a synthetic AIN-93M diet that did not contain NA. After acclimatization followed by fasting for 16 h, Fe (II) or NA-Fe (II) and ^59^Fe were administered to 8-week-old mice. Blood, spleen, liver, kidney, and small intestine samples were collected after 0.5 h, 2 h, and 5 h for analyses (Fig. 5, *A*–*B*). In the small intestine, the ^59^Fe count was markedly higher than that detected in the other tissues (Fig. 5, *A*–*B*). There was no significant difference in ^59^Fe counts in the issues between Fe (II) and NA-Fe (II) administrations at 0.5 h and 2 h (Fig. 5, *A* and *C*). However, 5 h after administration, the kidney and spleen tissues of mice administered with NA-Fe(II) showed significantly higher ^59^Fe counts than those administered with Fe(II) (Fig. 5*D*). Results obtained in similar experiments performed in the same manner but without fasting were similar to the results obtained with fasting (Fig. S5). Hemoglobin (Hb) levels in the whole blood were also measured in parallel. NA-Fe (II) induced a slight increase in Hb concentration 5 h after administration. Fe (II) led to a small decrease in Hb concentration, which was caused by fasting (Fig. 5*E*).

### Iron and NA concentrations in mouse intestinal sections upon administration of ^59^Fe (II) or NA-^59^Fe (II)

**Figure 6:**
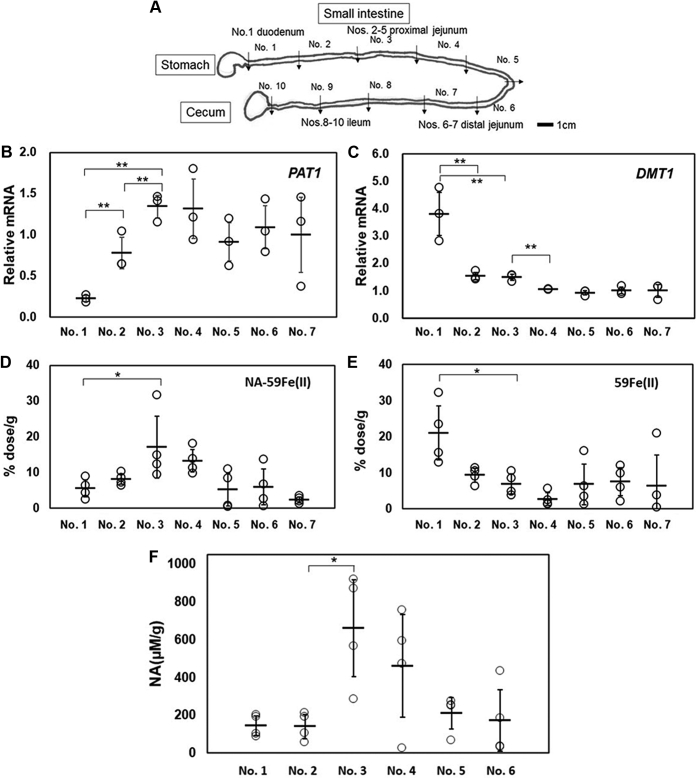
**Relative expression levels of *PAT1* and *DMT1*,**^**59**^**Fe and NA contents in various sections of mouse intestine.** (*A*) mouse small intestine was divided into ten sections (sections numbered 1–10) that were defined as the duodenum (section 1), proximal jejunum (sections 2–5), distal jejunum (sections 6–7), and ileum (sections 8–10). (*B*) mRNA levels of *PAT1*, (*C*) *DMT1* as determined by qRT-PCR in the duodenum (section. 1), proximal jejunum (sections 2–5), and distal jejunum (sections 6–7). The relative expression levels were calculated by setting the average values obtained for section 1 to 7 (n = 3–4, ∗∗*p* < 0.01, ∗*p* < 0.05). Uptake of ^59^Fe was determined by radioactivity measurements in the upper intestine sections 1 to 7 30 min after oral administration of NA-^59^Fe(II) (*D*) or ^59^Fe(II) (*E*) (n = 4, ∗*p* < 0.05). *F*, amounts of NA obtained from each section of the small intestine. Quantification of NA levels in these extracts was performed by quantitative analysis with LC-MS after FMOC conversion. (n = 4, ∗*p* < 0.05).

To gain further insight into the distribution of PAT1 expression in mice, we dissected small intestines into ten sections (sections 1–10 in Fig. 6*A*) (36) and examined *PAT1* and *DMT1* (free Fe(II)-uptake transporter) gene expression in each section. Quantitative RT-PCR analysis revealed that *PAT1* was abundantly expressed in the jejunum (sections 2–7) and was poorly expressed in the duodenum (Fig. 6*B*). *DMT1* displayed the highest mRNA expression in the duodenum (section 1; a 3-cm fragment from the pylorus) (Fig. 6*C*). ^59^Fe isotope was measured in each section for the duodenum (section 1), proximal (sections 2–5), and distal jejunum (sections 6–7) 0.5 h after NA-^59^Fe (II) administration. The highest level of iron was noted in the proximal jejunum (section 3) (Fig. 6*D*). Free ^59^Fe uptake by DMT1 was dominant in the duodenum (section 1) (Fig. 6*E*). These findings agreed with the relative levels of *PAT1* (Fig. 6*B*) but not those of *DMT1* mRNA (Fig. 6*C*). These results indicated that the NA-Fe(II) complex is absorbed by PAT1 mainly in the proximal jejunum, while free Fe (II) absorption by DMT1 takes place at the duodenum (8, 9).

The detection of NA and NA-Fe (II) complex in the intestinal sections was performed by TLC. The compound that was evident as spots after the application of ninhydrin was extracted and identified as NA by liquid chromatography–mass spectrometry (LC-MS) analysis after converting to a 9-fluorenylmethyloxy-carbonyl (FMOC) derivative (Fig. S6). TLC analyses of free ^59^Fe and NA-^59^Fe (II) detected by autoradiography revealed bands with an Rf value of 0 and 0.67, respectively (Fig. S7*A*). Extracts from the mouse upper small intestine (sections 1–6) collected 0.5 h after oral administration of NA-^59^Fe (II) also were analyzed by TLC. In the duodenum (section 1), a faint spot appeared at the origin corresponding to free ^59^Fe (Fig. S7*B*). Amounts of NA obtained from each section of the small intestine after NA-^59^Fe (II) administration were quantified using FMOC derivatization with LC-MS. The highest concentrations of NA were detected in the proximal jejunum, in sections 3 to 4 (Fig. 6*F*), consistent with the counts of ^59^Fe detected by autoradiography on TLC (Fig. S7*B*).

## Discussion

The results of this study reveal that NA forms an NA-Fe (II) complex that enhances iron uptake by the proton-coupled amino acid transporter PAT1. The NA-induced iron uptake occurs in the apical membrane of Caco-2 cells and likely in the jejunum of the small intestine. Iron uptake by PAT1 has been reported to be greatest in the upper regions of the intestine including the duodenum and the proximal jejunum (4). Based on these findings, we propose a new mechanism for iron uptake in mammals in which dietary NA complexed with Fe (II) is efficiently absorbed by the amino acid transporter PAT1, which also participates in iron uptake in the mammalian intestine. PAT1 is a member of the SLC proteins in eukaryotes. PAT1 was first identified as a lysosomal amino acid transporter for neutral amino acids (37). The hPAT1 human ortholog, which was first cloned from a Caco-2 cell-derived cDNA library, has 476 amino acids, encompassing nine putative transmembrane domains. The hPAT1 protein transports glycine, l-alanine, l-proline, α-amino isobutyrate, γ-aminobutyrate (28), and certain drugs (38). Quantitative structure–activity relationship analyses of substrates for hPAT1 have been reported (39). For example, bulky substrates bearing a six-membered ring can be efficiently transported by hPAT1. The results suggest that hPAT1 transports various amino acids with a relatively loose recognition of substrate bulkiness, implying that either linear NA or a bulkier NA-iron complex could be substrates of hPAT1. However, further experiments are necessary to elucidate the mechanism of the uptake of amino acid–metal complexes by hPAT1. In the present study, *hPAT1*-knockdown of Caco-2 cells led to a marked reduction in NA-^59^Fe (II) absorption but did not induce significant changes in absorption levels of inorganic iron. The amount of iron uptake by Caco-2 cells (Fig. 1*C*) was comparable between NA-Fe and free Fe. The amount of the free iron transported by DMT1 corresponded to that absorbed by a physiological intestinal epithelial cell (8, 9). The results of knockdown experiments (Fig. 4) suggested that most of the NA-Fe uptake was due to PAT1 activity, implying that iron taken up through this route is biologically relevant.

The present and previous data indicate that iron is likely taken up in the form of NA–iron complex in plants and mammalians. Presently, NA-Fe (II) increased the iron content in Caco-2 cells in a dose-dependent manner. This finding could explain previous findings that ferritin is highly expressed in Caco-2 cells to trap the absorbed iron at higher concentrations of NA-Fe(II) (27, 40). Activation of *OsNAS*, which encodes the rice NA-synthase enzyme (41), leads to increases in Fe concentrations in the leaves and seeds of rice (42, 43, 44). Furthermore, anemic mice fed on *OsNAS2*/*OsNAS3*-transgenic rice for 2 weeks showed normal levels of hemoglobin and hematocrit (45, 46). As shown in Figure 5*E*, measurements of hemoglobin contents during a 5-h fasting period after administration of NA-Fe (II) or Fe (II) revealed the induced increase and decrease in Hb concentrations, respectively. The present and prior data suggest that NA and PAT1 have biological roles in iron uptake in the mammalian intestine, particularly for the uptake of iron from plant-based diets. Further studies on the biological roles of PAT1 would require detailed *in vivo* experiments, which would benefit from generation of a PAT1-knockout mouse model.

It is commonly believed that iron absorption mostly occurs in the duodenum, where DMT1 is highly expressed and transports inorganic iron. Presently, in addition to DMT1, Fe (II) was efficiently absorbed in a complex with NA by PAT1 in the proximal jejunum. Free NA in the proximal intestine also plays a role in iron uptake as the affinity of NA for Fe(II) was very high with an association constant *K*_a_ of 1.3 to 6.3 × 10^12^ M^−1^ at pH 7.0. In aqueous solutions, NA bound Fe(II) at a pH ranging from 6.0 to 9.0 (20). The resulting NA-Fe (II) complex was stable in the order of pHs 8.0, 7.4, and 6.0 (Fig. 1*B*). Therefore, we hypothesize that free iron, once liberated from NA (or other chelators) due to low pH in the stomach, is readily chelated again by NA and absorbed by PAT1 in the proximal jejunum (Fig. 6). Clinical observations in patients that have undergone intestinal resection demonstrate the importance of the jejunum and other portions of the intestine in iron uptake. In a previous study, long-term survivors (more than 6 months) following pancreatoduodenectomy, in which the encircling loop of the duodenum together with the head of the pancreas was excised, had low ferritin levels (one-third of the levels observed in control subjects), but normal total iron levels in the serum, and none of the patients suffered from iron deficiency anemia (47). These observations suggest that for iron absorption in the small intestine, both the duodenum and jejunum are indispensable.

*In vivo*, free iron elicits the Fenton reaction, which generates hydroxyl radicals and leads to serious cell damage, which sometimes causes ferroptosis (48). However, iron mainly exists as an organic complex or a protein-conjugated form in mice and humans. NA is also reported to act as an iron scavenger to protect cells from the iron-mediated damages in plants (20). Recently, the Fe-free forms of closely related phytosiderophores (mugineic acids, MAs) were reported to decrease iron contents in murine serum, spleen, and liver, highlighting the potential of MAs in chelation therapies for iron-overloaded patients (49). The present results show that PAT1 transports free NA, suggesting that NA is even more preferable for use in chelation therapies, as NA is much more abundant in plant foods than MAs, and thus is unlikely to show any side effects upon oral administration. Iron deficiency in humans still poses a serious threat. For example, anemia often affects productivity and even causes serious health defects, including impaired cognitive development in children, a weakened immune system, and an increased risk of morbidity. An intestine-specific Dmt1 knockout mouse model was recently reported (50). This mouse model could be used to evaluate the role of the NA-mediated iron uptake in the human diet. Moreover, its combined use with the PAT1 knockout mice would clearly reveal the involvement of hPAT1 in transporting the NA–iron complex.

The present findings provide data that could inform significant advancements in the treatment of iron deficiency. NA supplied as a part of daily plant diet could potentially solve the problem by enhancing iron absorption, because iron supplements sometimes result in reduced absorption of iron in the small intestine. To achieve this goal, further mechanistic studies including the identification of the NA-Fe exporter on the basal side and the metabolic pathways regulating NA are necessary.

## Experimental procedures

### Caco-2 monolayer Fe transport assay

Caco-2 cells purchased from ATCC (Manassas, VA, USA) were routinely cultured in T75 culture flasks in high-glucose Dulbecco’s modified Eagle’s medium (DMEM; Nacalai Tesque, Kyoto, Japan) supplemented with 10% fetal bovine serum (Sigma-Aldrich, St Louis, MO, USA) and 1% penicillin/streptomycin (Nacalai Tesque) in a humidified incubator at 37 °C in a 5% CO_2_ atmosphere. The medium was changed every 2 or 3 days. Cells were passaged using 0.25% trypsin (Nacalai Tesque) at 37 °C. Cells were seeded (0.7 × 10^5^ cells/0.4 ml) in wells of Millicell 24-well cell culture plates (Merck Millipore, Billerica, MA, USA). On days 21 to 25 after seeding, cell monolayers were used for Fe or NA-Fe uptake experiments. Transport assays were essentially conducted as previously described (51). Caco-2 monolayers showing transepithelial electrical resistance values >700 Ω/cm^2^ as measured with Millicell inserts (Merck Millipore) were used in the experiments. Culture medium on both sides was removed by aspiration and the monolayers were washed twice with transport buffer (TB) that consisted of Hank’s balanced salt solution (Nacalai Tesque) and 10 mM HEPES (Nacalai Tesque), pH 7.4. The monolayers were preincubated for 15 min at 37 °C after adding 0.4 ml and 0.8 ml of TB buffer at the apical and the basolateral side, respectively. After removal of the medium, 0.4 ml of TB buffer adjusted to pH 6.0, 7.4, or 8.0 containing 1 mM sodium l-ascorbic acid (Sigma-Aldrich) and 100 μM free ^59^Fe(II) (FeSO_4_-7H_2_O) (Wako, Osaka, Japan), which consisted of 1 to 2 μM ^59^FeSO_4_ or ^59^FeCl_3_ (PerkinElmer Japan, Kanagawa, Japan) or NA-^59^Fe(II) (1 mM NA:100 μM ^59^Fe, 10:1) was added to the apical side. One milliliter of TB buffer was added to the basal side. NA was chemically synthesized as previously described (52) or was purchased from T. Hasegawa Co (Tokyo, Japan). The NA-Fe(II) complex was prepared as previously described (22, 53). After a 30-min incubation, the buffer on the apical side was aspirated, while that on the basal side was kept undisturbed. After washing the cells twice with 0.4 ml of TB buffer containing 5 mM EDTA, 0.4 ml of double-distilled water was added, and the monolayers were collected into a tube using a cell scraper (Greiner Bio-One, Kremsmünster, Austria). Cellular Fe concentrations were determined by measuring ^59^Fe radioactivity using a Cobra II Auto-Gamma Counter Model 5003 auto-well gamma counter (Packard, Downers Grove, IL, USA).

### RNAi-mediated suppression of hPAT1 expression in Caco-2 cells

Three pre-miRNA sequences for *hPAT1* were designed using an RNAi designer (Invitrogen, Carlsbad, CA, USA) for nucleotide sequences 463 to 483, 907 to 927, and 1608 to 1628 of the 5786-bp *hPAT1* mRNA (NM_078483) (28). The primer sequences are listed in Table S1. Vectors were generated as previously described (54). Complementary DNA oligos (TaKaRa Bio, Kyoto, Japan) were annealed to generate double-stranded oligos that were cloned into a linearized pcDNA 6.2-GW/EmGFP-miR vector (Invitrogen) using T4 DNA ligase. Negative microRNA (Neg-miRNA) control plasmid was included in the Block-iT-Pol II miR RNAi Expression Vector Kit. All vectors were transformed into One Shot TOP10 Chemically Competent *Escherichia coli* (Invitrogen). Spectinomycin-resistant transformants were analyzed for the desired expression clones. The recombinant vectors were purified with a purification kit (QIAGEN, Valencia, CA, USA) and confirmed by sequencing (TaKaRa Bio). Caco-2 cells were transiently transfected with *hPAT1* RNAi vectors or Neg-miRNA in 24-well plates for 24 h using HilyMax (Dojindo, Kumamoto, Japan) according to the manufacturer’s protocol. The transfected cells were maintained in DMEM containing 10 μg/ml blasticidin (Invitrogen). The medium was refreshed every 3 to 4 days. After approximately 1 week, the transfected cells were transferred to 15-cm dishes. After culturing for 1 week, colonies were selected using a cloning ring (Corning, Corning, NY, USA). After another week of culture, fluorescent cells were selected from the medium that contained 30 μg/ml blasticidin and were transferred to six-well plates. The cells were used for the qRT-PCR and monolayer transport assays. Primers for qRT-PCR are listed in Table S1. For western blotting, the two lines with *hPAT1* RNAi and control Caco-2 cells were lysed in RIPA buffer (Thermo Fisher Scientific, Waltham, MA, USA) containing complete EDTA-free Protease inhibitor (Roche, Mannheim, Germany). The crude lysates were sonicated for 2 s three times and centrifuged at 14,000*g* for 10 min. Protein concentration was determined using a BCA Protein Assay Kit (Thermo Fisher Scientific). Protein lysates (30 μg) were heated at 100 °C for 5 min in 6× SDS sample buffer and loaded on 5 to 20% SDS-PAGE gel (SPG-520L, ATTO, Japan). The resolved proteins were transferred to a PVDF membrane. Immunoblotting was performed using rabbit antibody to hPAT1 (Aviva System Biology, CA, USA) or mouse antibody to glyceraldehyde 3-phosphate dehydrogenase (GAPDH; Abcam, Cambridge, UK) at 4 °C overnight. Horseradish peroxidase–conjugated rabbit or mouse secondary antibody (GE Healthcare, Arlington Heights, IL, USA) was then applied. Reactive proteins were detected using Amersham ECL Prime reagent, and images were obtained using Amersham Imager600 (GE Healthcare).

### Expression of hPAT1 in *Xenopus* oocytes

For ectopic *hPAT1* expression in oocytes, human small intestine total RNA (Clontech, Mountain View, CA, USA) was reverse-transcribed with SuperScript III First Strand Synthesis Super Mix (Invitrogen). The open reading frame of *hPAT1* (NM_078483) was amplified from the cDNA by PCR using the forward primer 5′-CGGAATTCACCATGTCCACGCAGAGACTTC-3′ and reverse primer 5′-GGCTCTAGATCCCTACTATATGAAGGCACAG-3′. PCR products were purified using the QIAquick PCR purification kit (Qiagen) and were inserted into the *EcoR*I and *Xba*I sites of the pTNT *Xenopus* oocyte expression vector (Promega, Madison, WI, USA). Constructs were verified by sequencing. The hPAT1/pTNT vector was linearized with *Not*I and cRNA transcription was performed using the SP6 mMESSAGE mMACHINE kit (Ambion, Austin, TX, USA). The cRNA solution (1 μg/μl, 25 nl) was injected into oocytes, which were incubated in ND96 buffer (pH 7.6) containing NaCl (96 mM), KCl (2 mM), CaCl_2_ (1.8 mM), MgCl_2_ (1 mM), and HEPES (5 mM) at 16 °C for 3 to 4 days. Oocytes injected with water were used as a negative control. For ectopic *hPEPT1*or *hDMT1* expression in oocytes, the genes were amplified by PCR using specific primers (Table S1) and inserted into the same pTNT vector used for hPAT1. For immunofluorescence microscopy, cryo-sections (16 μm) from oocytes (55) were treated with primary monoclonal antibodies to hPAT1 (OriGene Technologies Inc, Rockville, MD, USA) or PMCA ATPase (Thermo Fisher Scientific) in 5% bovine serum albumin in PBS-Tween (BSA/PBST) at 25 to 27 °C for 1 h, washed three timed in PBST, and probed with anti-rabbit Alexa 546 (Molecular Probes, Eugene, OR, USA) for PAT1 and anti-mouse Alexa 488 (Molecular Probes) for PMCA ATPase in 5% BSA/PBST for 1 h. After washing three times, the samples were mounted using VECTASHIELD (Vector Laboratories Inc, Burlingame, CA, USA). Microscopy analysis was performed with a Fluoview FV100 confocal laser scanning biological microscope (Olympus, Tokyo, Japan).

### Electrophysiological assays in *Xenopus* oocytes

The analysis was performed essentially as reported previously (31, 53). *X. laevis* (Kato-S Science, Chiba, Japan) oocytes were voltage-clamped at −60 mV with an OC-725C oocyte clamp (Warner Instruments, Hamden, CT, USA) and were placed in an open chamber with continuous perfusion of ND96 buffer (pH 6.0) or ND96-containing substrates. Steady-state currents were obtained after the addition of NA or NA-Fe(II) complex at a concentration of 0, 10, 20, 50, 100, or 200 μM in 10 mM MES/Tris buffer (pH 6.0) (31, 33). Acquisition and all subsequent analyses were performed using p-Clamp 10 (Molecular Devices, Sunnyvale, CA, USA). For the inhibition experiments, 1 or 5 mM 5-HTP (LKT Laboratories, St Paul, MN, USA) was used for electrophysiological assays with 1 mM NA-Fe(II).

### RNA extraction and qRT-PCR analysis

RNA was extracted with the RNeasy Plus Mini Kit (QIAGEN). Sections of mouse small intestines that had been stored at −80 °C were ground in a mortar with Sepasol-RNA I Super G (Nacalai Tesque), and RNA was prepared using the phenol/chloroform extraction method. RNA quality was confirmed by spectrophotometry (NanoDrop Technology, Wilmington, DE, USA). RNA was reverse-transcribed with the Superscript III RT Kit (Invitrogen) according to the manufacturer’s instructions. Quantitative RT-PCR was performed on a CFX96 Real-Time System (Bio-Rad, Hercules, CA, USA) using SsoAdvanced Universal SYBR Green Supermix (Bio-Rad). Reactions (10 μl) were run in triplicate using the following thermal incubations: 3 min at 95 °C, 40 cycles of 10 s at 95 °C followed by 30 s at 60 °C, and 10 s at 95 °C. Table S1 lists the primer sequences. The relative efficiency of each primer set was determined from standard curves generated using tenfold dilutions of cDNA. The mRNA levels of each gene were normalized to those of *GAPDH*.

### Animals

Male ICR mice purchased from CLEA Japan, Inc were acclimatized and maintained on standard feed or synthetic AIN-93M feed (CE-2, CLEA Japan, Inc, Tokyo, Japan) with free access to water. All experiments were approved by the Animal Care and Animal Ethics Committees of Kyoto Pharmaceutical University (No. ABCH-19-014). All animals were maintained in accordance with the committee guidelines for the care and use of laboratory animals.

### Determination of iron content and blood Hb levels in mouse tissues

Male 7-week-old mice were fed synthetic AIN-93M for 1 week. The mice were divided into two groups. Those in the first group were fasted for 16 to 20 h. Those in the second group were not fasted. Oral administration by intragastric gavage was performed using a stomach tube with 0.1 ml of 3.3 mM Fe(II) alone or with an additional 33 mM NA (NA/Fe(II) ratio = 10:1) containing ^59^Fe isotope in PBS with 1 mM ascorbic acid. After 0.5, 2, or 5 h, blood and tissue samples were collected and analyzed by measuring ^59^Fe radioactivity using an auto-well gamma counter. Blood Hb levels were measured using QuantiChrom Whole Blood Hb Kit (DWHB-250; BioAssay Systems, Hayward, CA, USA). Thirty minutes after administration, mouse small intestines were divided into ten sections (36). Iron levels in the duodenum (section 1), proximal jejunum (sections 3–5), and distal jejunum (sections 6–7) were measured. The lumen sides of these intestinal samples were washed by PBS containing 5 mM EDTA and weighed. Isotope contents were assessed by measuring ^59^Fe radioactivity using an auto-well gamma counter.

### Determination of NA levels in small intestine

Thirty minutes after NA-Fe (II) administration, the lumen sides of small intestine sections 1 to 6 were washed by PBS containing 5 mM EDTA and weighed. Each section was placed in 1 ml of water and sonicated on ice with a model UD-211 ultrasonic disruptor (TOMY SEIKO Co, Tokyo, Japan) twice for 3 s each time. Samples were centrifuged at 15,300*g* for 10 min at 4 °C and the supernatant was collected. After the addition of 150 μl of water and 850 μl of acetonitrile to 10 μl of the supernatant, the solution was mixed and centrifuged at 2300*g* for 10 min. The supernatant was collected (900 μl), concentrated for 50 min with a centrifugal evaporator, and brought up to 100 μl with water. NA was quantitated using FMOC derivatization (Fig. S6). To 20 μl NA solution, 10 μl of 5 mM EDTA, 25 μl of 0.4 N borate buffer (pH 10.2), and 5 μl of FMOC-Cl in acetonitrile (Agilent Technologies, Santa Clara, CA, USA) were sequentially added. The mixture was vigorously mixed by vortexing at 20 °C. After the reaction, 10 μl of 50 mM 3-amino-1-propanol was added to terminate the derivatization, and 30 μl of distilled deionized water was added (total 100 μl). The samples were subjected to LC-MS on a LCMS-8030 system (Shimadzu, Kyoto, Japan) equipped with a 2.1 i.d. × 150 mm COSMOSIL 5C18-AR-II column (Nacalai Tesque). The injection volume was 10 μl. The mobile phase consisted of solvent A, MilliQ water containing 0.1% (v/v) formic acid, and solvent B, acetonitrile containing 0.1% (v/v) formic acid. The linear gradient used was as follows: 0 to 20 min, 10 to 65% B and 20 to 21 min 65 to 10% B. Electrospray ionization (positive ion mode) MS was operated in the selected ion monitoring mode to observe at *m/z* 526.2 [NA-FMOC + H] ^+^. Nebulizer gas pressure was set at 3 L/min. DL (Desolvation Line) and heat block temperatures were set at 250 °C and 400 °C, respectively. A calibration curve of derivatized NA was obtained using authentic sample within 0.0977 to 6.25 μM.

### TLC analysis of NA

High-performance TLC plates with cellulose on aluminum sheet (Merck, Darmstadt Germany) were employed for detection of NA and NA-Fe (II) complex. Freshly prepared PBS solution of 16.5 mM NA alone or with an additional 16.5 mM FeSO_4_ was deposited on the TLC start line at a volume of 1 μl and developed to a distance of approximately 5 cm. NA was detected by staining with 0.3 g ninhydrin (Nacalai Tesque), 3 ml acetic acid 3 ml, and 100 ml n-butanol. Thirty minutes after administration of 0.4 ml of 1 mM NA-^59^Fe(II), Caco-2 cells were washed twice with TB buffer containing 5 mM EDTA, collected in 0.4 ml of water, supplemented with 50 μl of 1 M Tris buffer (pH 8.0), and vortexed. Cell extracts (1 μl) were applied to the cellulose TLC plate. Thirty minutes after the administration of NA-^59^Fe (II), 200 μl Tris-HCl buffer (pH 8.0) was added on small intestine sections 1 to 6. The mixture was vortexed and centrifuged at 13,000 rpm for 10 min. Each supernatant (1 μl) was applied to the TLC plate and developed with a solvent of acetonitrile: water: acetic acid at a ratio of 1:1:0.2. After developing and drying the TLC plate, ^59^Fe was detected by a Typhoon Biomolecular Imager (GE Healthcare Bio-Science Corp, Marlborough, MA, USA), and ninhydrin staining was performed.

### Statistical analysis

Statistical analysis was carried out using Excel software (Microsoft, Redmond, WA, USA). The values are expressed as mean ± standard deviation (SD), as calculated by STDEVP. Groups were compared by Student’s *t*-test. *p*-values < 0.05 indicated significant differences.

## Data availability

The mRNA and protein sequences presented in this article are registered in the NCBI database with the following accession numbers; PAT1 (human, NM_078483; mouse, NM_153139), PAT2 (human, NM_181776), PAT3 (human, NM_181774), PAT4 (human, NM_001286139), DMT1 (human, NM_000617; mouse, NM_001146161), PEPT1 (human, NM_005073), GAPDH (human, NM_001289745; mouse, NM_001289726), and ZmYS1 (*Zea mays*, AF186234).

All remaining data are contained within the article.

## Conflict of interest

The authors declare that they have no conflicts of interest with the contents of this article.
