# Supplementary material for: Iron uptake mediated by the plant-derived chelator nicotianamine in the small intestine
Source: J Biol Chem. 2020 Dec 25;296:100195. doi: 10.1074/jbc.RA120.015861 (PMC7948497; doi:10.1074/jbc.RA120.015861)
Supplement: Supplementary Figures and Tables [file mmc1.pdf]

Iron uptake mediated by the plant-derived chelator nicotianamine in the small intestine

Yoshiko Murata[1,*], Masami Yoshida[1], Naho Sakamoto[1], Shiho Morimoto[1], Takehiro Watanabe[1] and Kosuke Namba[2]

[1]Bioorganic Research Institute, Suntory Foundation for Life Sciences, 8-1-1 Seikadai, Seika-cho, Soraku-gun, Kyoto 619-0284, Japan
[2]Graduate School of Pharmaceutical Science, Tokushima University, Shomachi 1-78-1, Tokushima, 770-8505, Japan

[*]Corresponding author:
Yoshiko Murata Ph.D.
Bioorganic Research Institute, Suntory Foundation for Life Sciences
8-1-1 Seikadai, Seika-cho, Soraku-gun, Kyoto 619-0284, Japan
Tel: +81-50-3182-0705
Fax: +81-774-98-6292
E-mail: murata@sunbor.or.jp

File Name: Supporting Information

Description: 7 Supporting Figures and 1 Supporting Table

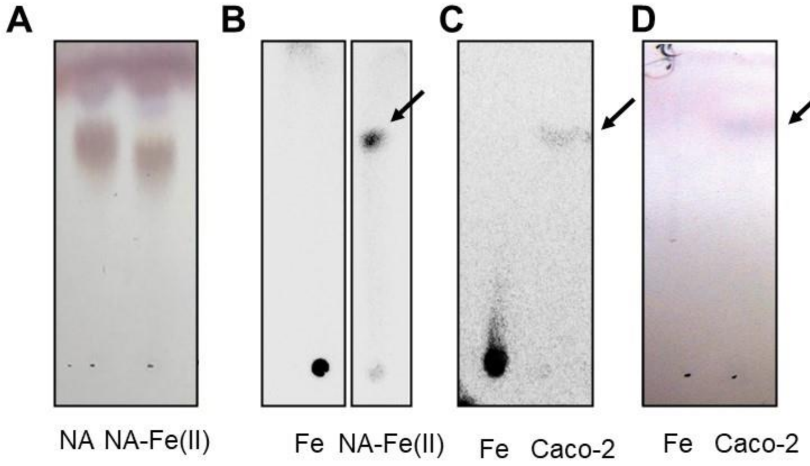

Figure. S1 Thin-layer chromatography (TLC) analysis of NA-Fe (II) complex. (**A**) Synthetic NA (16.5 mM, 0.5 µL) and NA-Fe(II)(NA/Fe=1:1,16.5 mM, 0.5 µL) containing 1mM ascorbic acid were detected by ninhydrin staining (detection of amine). **(B**) 1 µL of 3.3 mM ^{59}Fe and NA-^{59}Fe(II) were detected by autoradiography on TLC. (**C**, **D**) Caco-2 cells were extracted at 30min after administration 1 mM NA-^{59}Fe(II) and the extract was applied on cellulose TLC with authentic $^{59}FeSO_4$. ^{59}Fe was detected by autoradiography on TLC (**C**) and the amino acid was detected with ninhydrin staining (**D**). The bands corresponding to NA-Fe(II) (Rf=0.67) were indicated with arrows. All TLC plates were developed with MeCN:H_2O:AcOH=1:1:0.2.

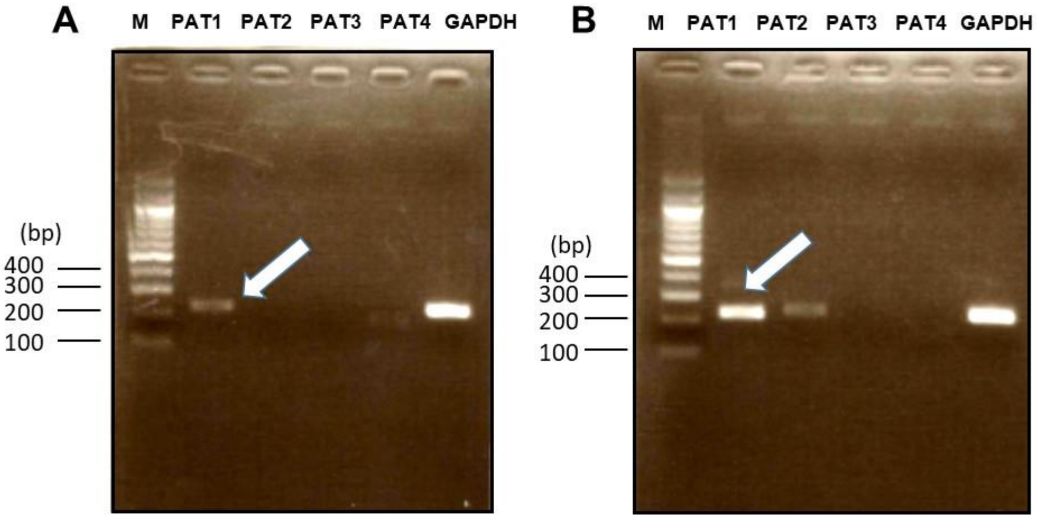

Figure S2. mRNA expression of four PAT proteins (PAT1–PAT4) in human intestine and Caco-2 cells evaluated by RT-PCR. RT-PCR from (**A**) human intestine and (**B**) Caco-2 cell RNA of PAT1-4. RT-PCR analysis of the human intestinal and Caco-2 cells revealed that only primers targeting *PAT1* yielded a 228-bp PCR product, indicated by the arrows (Fig. S2A, B). The RT-PCR of targeting human *GAPDH* as control yielded a 238-bp product.

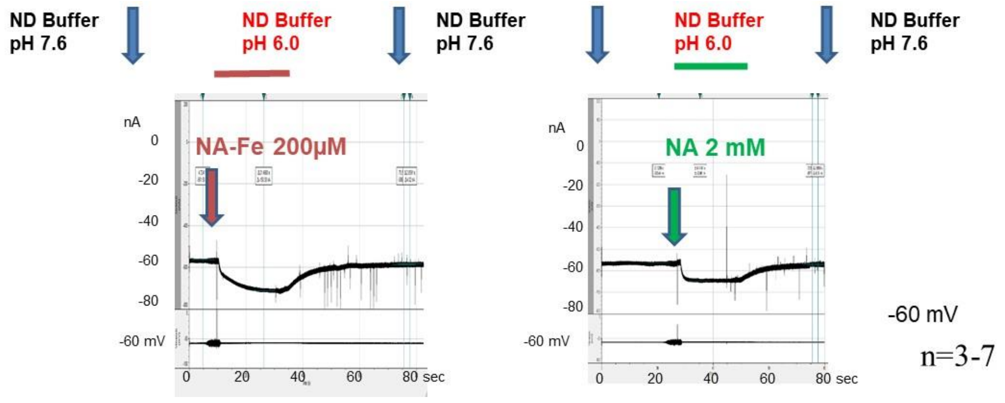

Figure S3. Current changes were recorded after the addition of 2 mM nicotianamine (NA) or 200 µM NA-Fe(II). NA/Fe molar ratio = 10:1. *Xenopus laevis* oocytes were voltage-clamped at -60 mV with an OC-725C oocyte clamp (Warner Instruments, Hamden, CT, USA) and were placed in an open chamber with continuous perfusion of ND96 buffer (pH 6.0) or ND96-containing substrates. Steady-state currents were obtained after the addition of NA or NA-Fe(II) complex in 10 mM MES/Tris buffer (pH 6.0). Acquisition and all subsequent analyses were performed using p-*Clamp 10* (Molecular Devices, Sunnyvale, CA, USA).

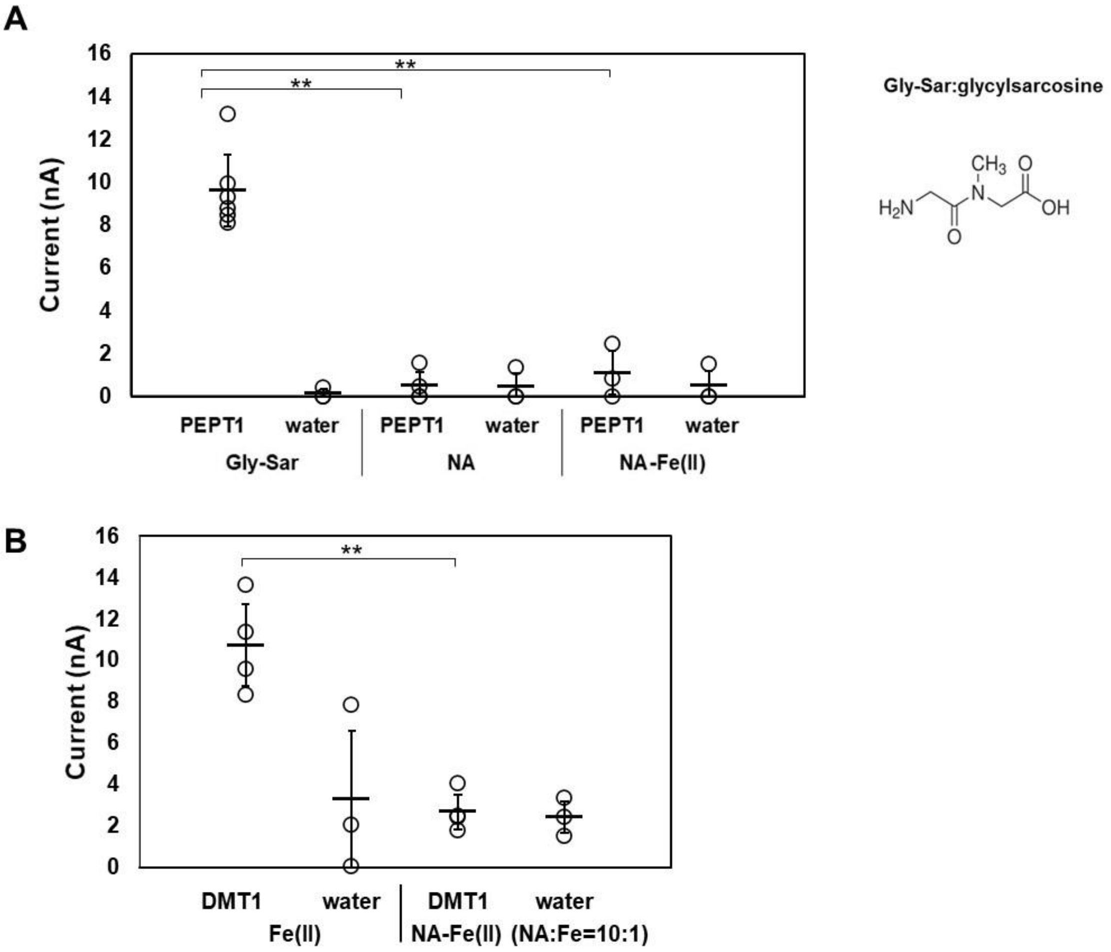

Figure S4. Electrophysiological assay of *Xenopus laevis* oocytes expressing hPEPT1 and hDMT1. (**A**) Oocytes were injected with *hPAT1* cRNA or water (as negative control), held at –60 mV during current recording, and superfused with sample buffer (pH 6.0) containing 500 µM glycylsarcosine (Gly-Sar) (n = 6) and 500 µM NA-Fe (NA/Fe molar ratio = 2:1) (n = 3). (**B**) Oocytes were injected with *hDMT1* cRNA or water, and superfused with sample buffer (pH 6.0) containing 50 µM Fe or 50 µM NA-Fe(II) (NA/Fe molar ratios = 10:1) (n = 4) held at –60 mV. **$P < 0.01$

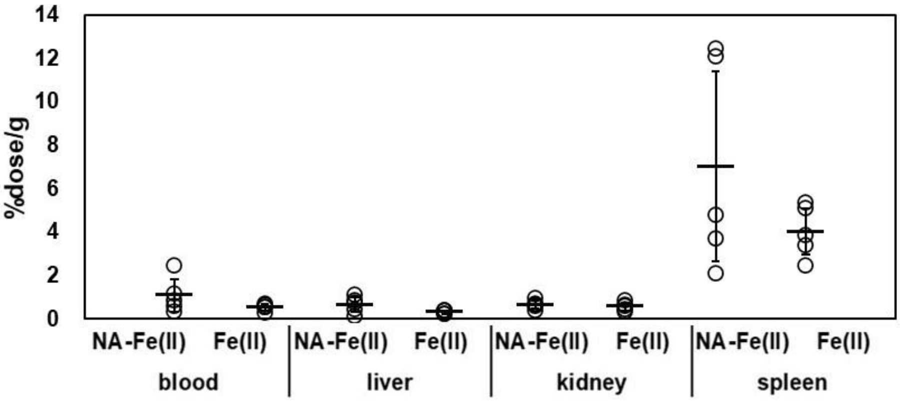

Figure S5. Comparison of iron absorption after oral administration of Fe and NA-Fe(II) without fasting. ^{59}Fe radioactivity was counted in the blood, liver, kidney, and spleen of mice at 5 h after oral administration of NA-^{59}Fe(II) or ^{59}Fe(II) (n = 7–9). The vertical axis is ^{59}Fe counts for each organ per gram weight that was expressed as the percentage of total activity of ^{59}Fe administered to mice.

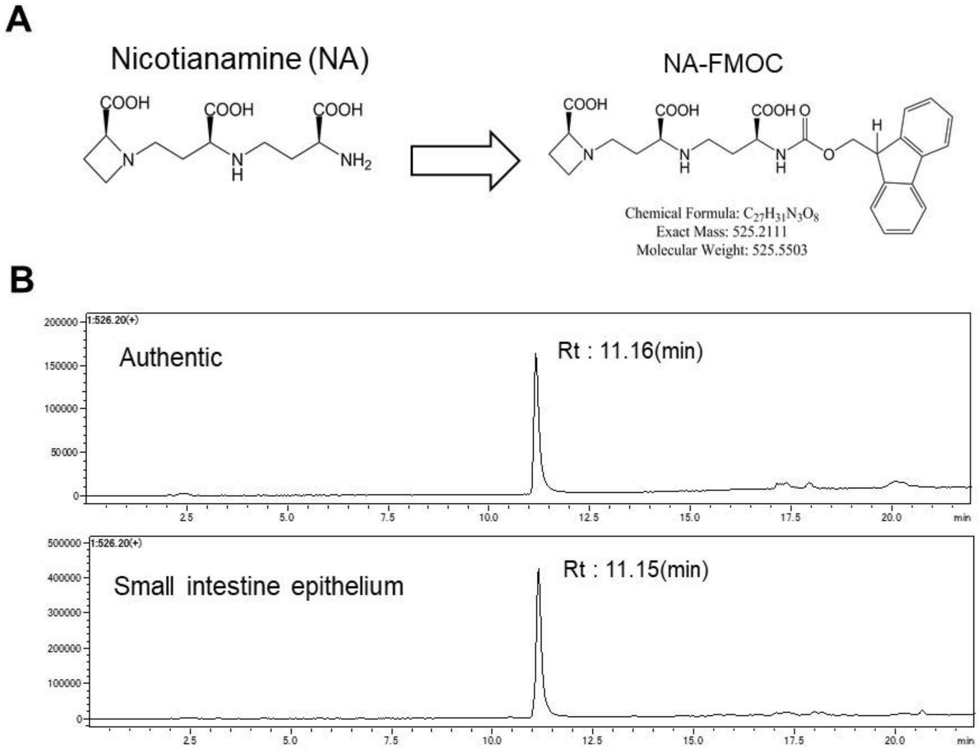

Figure. S6 Quantitative analysis of NA using 9-fluorenylmethyloxycarbonyl (FMOC) derivatization. Mass spectrometry was operated in the selected ion monitoring mode for observation at *m/z* 526.2 $[NA\text{-}FMOC + H]^+$ (**A**) and MS chromatograms of synthetic NA and the extract from small intestine were detected at a retention time (Rt) of 11.15 min (**B**). Analytical methods are described in the Experimental Procedures section in the manuscript.

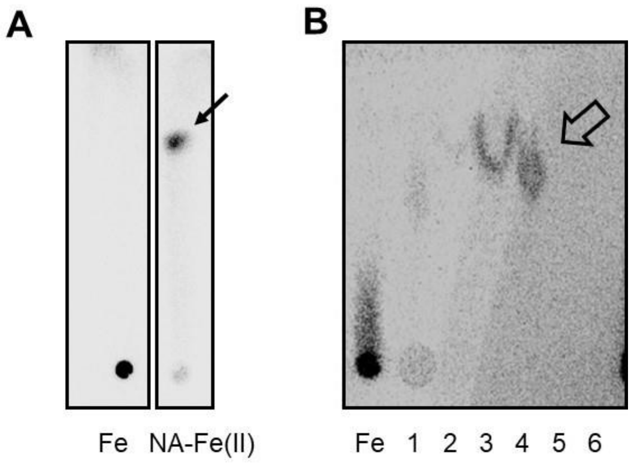

Figure. S7 Thin-layer chromatography (TLC) analysis of NA-Fe(II) complex in the small intestine. (**A**) ^{59}Fe and NA-^{59}Fe(II) (3.3 mM, 1 μL) were detected by autoradiography on TLC. The bands corresponding to NA-^{59}Fe(II) (Rf=0.67) are indicated by arrows. This panel, which is the same as Fig. S1B, is shown to make it easier to compare the bands corresponding to NA-^{59}Fe(II) in panels A and B. (**B**) Extracts from mouse upper small intestine (sections1-6) at 30 min after oral administration of NA-^{59}Fe(II) were applied to the cellulose TLC. ^{59}Fe was detected by autoradiography on TLC. The bands (Rf=0.6-0.7) are indicated by arrows. All TLC plates were developed with MeCN:H_2O:AcOH = 1:1:0.2.

Table S1. Primers used in the study

Primer sequences for *hPAT1* RNAi Caco-2 cells

miR Select Oligo ID	Forward (5' to 3')	Reverse (5' to 3')	Targeted regions (bp)
Hmi 460740	TGCTGTGAAGATGGGAGCATTGCTTGGTTTTGGCCACTGACTGACCAAGCAATTCCCATCTTCA	GTGAAGATGGGAGCATTGCTTGGTTTTGGCCACTGACTGACCAAGCAATTCCCATCTTCACAGG	1608-1628
Hmi 460741	TGCTGATGATCAGCAGGCTGATGGGAGTTTTGGCCACTGACTGACTCCCATCACTGCTGATCAT	GATGATCAGCAGGCTGATGGGAGTTTTGGCCACTGACTGACTCCCATCACTGCTGATCATCAGG	463-483
Hmi 460743	TGCTGATCATGACCAAGCTGACCAGCGTTTTGGCCACTGACTGACGCTGGTCATTGGTCATGAT	GATCATGACCAAGCTGACCAGCGTTTTGGCCACTGACTGACGCTGGTCATTGGTCATGATCAGG	907-927

Primer sequences for Quantitative RT-PCR

mouse	Forward (5' to 3')	Reverse (5' to 3')
GAPDH	CTGCACCACCAACTGCTTAG	GTCTTCTGGGTGGCAGTGAT
DMT1	TACCCATCCTCACGTTCACA	TTGATGGAGCAGACGATCAG
PAT1	ACCTGCTGAAAGGCAACATT	CTTCACCAGGATACCCATGC

human	Forward (5' to 3')	Reverse (5' to 3')
GAPDH	CCAGGTGGTCTCCTCTGACT	CCCTGTTGCTGTAGCCAAAT
DMT1	GAGTGGTTACTGGGCTGCAT	GCATGTCTGAGCCGATGATA
PAT1	ATGCTGGTCAGCTTGGTCAT	AACCATTCCAATGCCTTCAA

Primer sequences for RT-PCR

human	Forward (5’ to 3’)	Reverse (5’ to 3’)	bp
PAT1	AAGGCATTGGAATGGTTCTG	TGAAAAAGATCCCGATGGAG	228
PAT2	ACGTCCCTGCAGAAATCATC	GGCTCATGCCCTCTGAGTAG	237
PAT3	TACTGGGGACACTGGGCTAC	ACACTTGGGAGATGGCAAAC	186
PAT4	GGATGTAATGAGGCCCTTGA	TCCTAAAAGGCCAGTTCCAA	178
GAPDH	GAGTCAACGGATTTGGTCGT	TTGATTTTGGAGGGATCTCG	238

Primer sequences for cRNA of *Xenopus* oocytes

human	Forward (5’ to 3’)	Reverse (5’ to 3’)
PEPT1	GGCCCTCGAGATGGGAATGTCC AAATC (*Xho*I)	GGCCTCTAGATCACATCTGTTTC TGTG (*Xba*I)
DMT1	GGCCCTCGAGATGGTGCTGGGT CCTGA (*Xho*I)	GGCCCCATGGTTATTTAACGTAG CCAC (*Kpn*I)
PAT1	CGGAATTCACCATGTCCACGCA GAGACTTC (*EcoR*1)	GGCTCTAGATCCCTACTATATGA AGGCACAG (*Xba*I)
